# Utilizing citizen science data to rapidly assess changing associations between wild birds and avian influenza outbreaks in poultry

**DOI:** 10.1098/rspb.2024.1713

**Published:** 2024-09-25

**Authors:** Stephen H. Vickers, Jayna Raghwani, Ashley C. Banyard, Ian H. Brown, Guillaume Fournie, Sarah C. Hill

**Affiliations:** ^1^ Department of Pathobiology and Population Sciences, Royal Veterinary College, Hawkshead Lane, Hatfield AL9 7TA, UK; ^2^ Virology Department, Animal and Plant Health Agency, Surrey KT15 3NB, UK; ^3^ WOAH/FAO International Reference Laboratory for Avian Influenza, Swine Influenza and Newcastle Disease, Animal and Plant Health Agency, Surrey KT15 3NB, UK; ^4^ Ecosystems and Environment Research Centre, School of Science, Engineering and Environment, University of Salford, Greater Manchester, M5 4WT, UK; ^5^ Université de Lyon, INRAE,VetAgro Sup, UMR EPIA, Marcy l’Etoile, France; ^6^ Université Clermont Auvergne, INRAE, VetAgro Sup, UMR EPIA, Saint-Gènes-Champanelle, France

**Keywords:** HPAIV, spillover, viral incursion, disease vector, eBird

## Abstract

High pathogenicity avian influenza virus (HPAIV) is a rapidly evolving virus causing significant economic and environmental harm. Wild birds are a key viral reservoir and an important source of viral incursions into animal populations, including poultry. However, we lack a thorough understanding of which species drive incursions and whether this changes over time. We explored associations between the abundances of 152 avian species and outbreaks of highly pathogenic avian influenza (HPAI) in poultry premises across Great Britain between October 2021 and January 2023. Spatial generalized additive models were used, with species abundance distributions sourced from eBird. Associations were investigated at the species-specific level and across species aggregations. During autumn/winter, associations were generally strongest with waterbirds such as ducks and geese; however, we also found significant associations in groups such as non-native gamebirds and rapid change in species-specific associations over time. Our results demonstrate the value of citizen science to rapidly explore wild species as potential facilitators of disease incursions into well-monitored populations, especially in regions where viral surveillance in wild species is limited. This can be a critical step towards prioritizing targeted surveillance that could inform species-specific biosecurity measures; particularly for HPAIV, which has undergone sudden shifts in host range and continues to rapidly evolve.

## 1. Introduction

The emergence and rapid evolution of transboundary animal pathogens pose significant economic and zoonotic risks [1]. Pathogens that are transmissible by multiple wild-host species are particularly challenging to control, in part because the importance of each wild species as a reservoir or bridging host can vary seasonally alongside changes in species ecology. Host importance can also shift rapidly as a result of pathogen evolution or diffusion into new areas [2]. Continuously monitoring how different wild hosts contribute to disease spread is therefore essential for designing effective control measures and developing predictive disease transmission models [3]. However, this is often prevented by the high cost and difficulty of conducting widespread pathogen surveillance in wildlife.

Avian influenza virus (AIV) has among the widest geographic and host range of all transboundary animal diseases [4]. In 2020, the emergence of high pathogenicity AIV (HPAIV) subtype H5Nx clade 2.3.4.4b led to global disease outbreaks that profoundly impacted both the poultry industry and wild-bird populations, causing significant economic and environmental harm [5,6]. The main wild-host reservoirs of AIVs are ducks and other waterfowl (Order Anseriformes, family *Anatidae*), with infection often resulting in subclinical disease outcomes [7,8], although severe to lethal outcomes may be more common in some species [9]. However, recent outbreaks of H5Nx clade 2.3.4.4b have been characterized by higher observed mortality across a broader range of wild birds than previously observed, including widespread HPAIV infections detected in seabirds for the first time [10–12]. This suggests that relying on our knowledge of previous AIV outbreak ecology when responding to this outbreak is insufficient. Mass-mortality events attributed to H5 clade 2.3.4.4b have now been recorded in many groups, especially wildfowl (Order Anseriformes), shorebirds (suborders Charadrii and Scolopaci), seabirds (Orders Procellariiformes, Pelecaniformes, Gaviiformes and Podicipediformes, and Suborders Lari and Alcae) and birds of prey (families *Strigiformes*, *Accipitridae* and *Falconidae*) [12–15]. Challenge studies and wild-bird surveillance have also demonstrated that many additional species that have not suffered mass mortality can also carry HPAIV [6,16–18].

During annual disease outbreaks of highly pathogenic avian influenza (HPAI) in Great Britain since 2020, farm contact tracing and genetic analysis of viruses from infected poultry have strongly implicated wild birds as the likely primary source of repeated independent incursions, rather than farm-to-farm transmission [19]. However, our understanding of which of the many affected wild-bird species may contribute to such incursions, and whether this varies between outbreaks due to continued virus evolution, changes in wild avian species assemblages and abundance, and heightened premise biosecurity, remains relatively coarse [20,21]. Species that are known to contribute to the international spread of HPAIV (particularly wildfowl and gulls [20,22]) may not necessarily be sufficient to explain incursions onto farm premises. Instead, there may be additional species acting as local amplifiers of viruses, which could be critical in facilitating viral incursions. Even those groups that are more rarely found to be infected, such as passerines [23,24], could still contribute to incursions into poultry. For example, the House Sparrow *Passer domesticus* is frequently found on farms [25], can easily enter poorly secured housing because of its small size, and has been hypothesized to have a role as either a bridging host or a mechanical vector in introductions to poultry premises [26].

Understanding species-specific patterns of spillover from wild to domestic birds is difficult in part due to obstacles in effective monitoring of infection across the wide breadth of potential hosts. Surveillance of outbreaks in wild birds can be severely limited by financial constraints, detection and sampling of carcasses, and requirements for high biosecurity testing facilities [27]. Testing may, therefore, be heavily skewed towards species with overt clinical symptoms or for which carcasses are more easily detectable. For example, while all suspected kept-bird premise cases in Great Britain are tested, only a relatively small subset of reported wild-bird carcasses suspected of being HPAIV positive can be tested [28].

One way to potentially achieve better prioritization of wild-bird surveillance is by assessing associations between the abundance of species that could potentially facilitate viral incursions and confirmed infections in monitored populations such as premises housing domestic poultry or other captive birds (henceforth referred to collectively as ‘captive avian premises’). This is based on a reasonable assumption that there will be a generally higher incidence of cases where one or more species that facilitate incursion are at higher abundance. While this approach does not rely upon *a priori* knowledge of potential wild-host species, it does require an accurate understanding of spatio-temporal patterns of species abundance across the region of interest. Modelling spatio-temporal patterns of species abundance at national scales is complex because it requires a large amount of observational data and computationally intensive analyses [29]. However, citizen science initiatives alongside advances in computational power and novel analytical approaches that account for biases in semi-structured data collection procedures have enabled rapid advances in our understanding of species abundance and distributions. The citizen science initiative eBird is one such example of this, currently providing estimates of the full annual cycle distributions and abundances for >2000 avian species [30]. With these advances, it is now possible to rapidly assess the associations between the spatio-temporal distribution of wild-bird populations and disease outbreaks in poultry at larger scales than previously possible.

As many different wild-bird species may be involved in viral transmission, with varying levels of interaction with each other and captive avian premises, assessing species-specific potential as facilitators of virus incursion into captive flocks can be challenging. Using eBird-modelled species abundance estimates that are publicly available for a broad selection of wild-avian species, we investigate spatial associations between species-specific wild-bird abundance and patterns of HPAI disease cases in captive avian premises across Great Britain. The use of citizen science recording schemes such as eBird benefits from being non-reliant on any field- or laboratory-led surveillance in potential wild-viral hosts, and as such can be rapidly deployed against pathogens with wild reservoirs of infection in areas with limited wild-host testing capacity. Continued monitoring of associations between wild hosts and detected disease outbreaks in captive avian premises could prove useful as an early-warning sign of a possible virus host range expansion into a new wild species that is not currently subject to surveillance, and to help prioritize surveillance of species that may be facilitating incursions into captive birds and other well-monitored populations.

## 2. Methods

We used publicly available model-predicted abundance distributions of wild birds to explore spatial associations with HPAI cases in premises across Great Britain between 19 October 2021 and 20 January 2023. Species abundance predictions are produced using data from eBird, a global community science monitoring program administered by The Cornell Lab of Ornithology [30,31].

### (a) Data processing

Relative abundance distributions (RADs) were retrieved from eBird Status and Trends using R package ebirdst [31]. Following eBird terminology, ‘relative abundance’ is computed as the product of: (i) the probability of occurrence (0−1) and (ii) the species count conditional on occurrence. Where ‘occurrence’ is defined as the expected probability of encountering the species during a standardized eBird survey effort, and ‘count’ as the expected abundance that would be recorded when the species is present. Relative abundance is therefore independent of other species’ abundances in this context. These RADs provided estimates for the full annual cycle at weekly intervals, modelled on the year 2021, across a regular grid which we use at a resolution of 27 km^2^ (i.e. the lowest resolution available directly from ebirdst). Predicted RADs are derived from an ensemble modelling strategy based on the Adaptive Spatio-Temporal Exploratory Model (AdaSTEM [29]). For full RAD methodology, see Fink *et al*. [32]. RADs that include Great Britain are currently available for 256 species, with seasonal quality ratings assessed between 0 (lowest quality) and 3 (highest quality). We consider only those species with scores of 2 or 3 during the modelled period (to exclude predictions with low confidence) and a sum of relative weighted abundance scores within the relevant time period of >1 (to exclude uncommon and rare species). This produced a final pool of 152 species. Seasonal quality rating scores are assigned based on expert human review, whereby a rating of 0 implies predictions failed review and ratings of 1−3 correspond to decreasing levels of extrapolation and/or omission.

**Figure 1.**
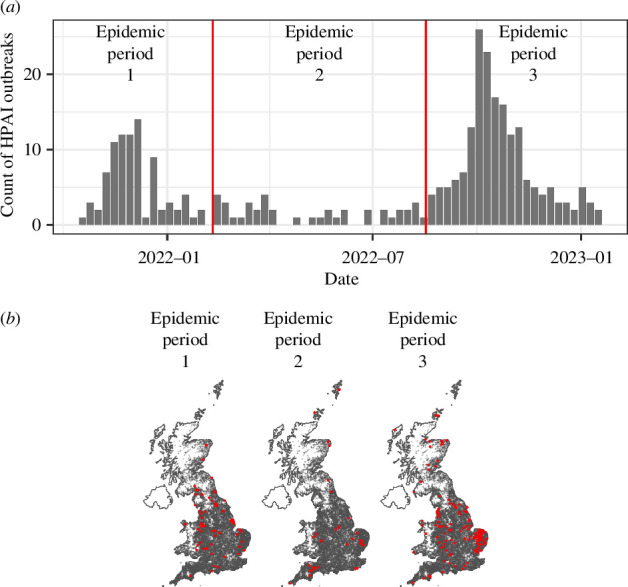
HPAI cases in captive avian premises in Great Britain between 19 October 2021 and 20 January 2023. (*a*) Epidemic curve split into three distinct EPs (EP1 = 19 October 2021–9 February 2022; EP2 = 10 February 2022–16 August 2022; EP3 = 17 August 2022–20 January 2023). Number of cases per period were 86, 40 and 186, respectively. (*b*) Map of cases (red points) against a map of premises (black points) as listed in the Great British Poultry Register (jittered here to maintain anonymity).

During our study period, there were 312 HPAI cases across captive avian premises in Great Britain (figure 1). Case data on infected premises housing captive birds was obtained from the Animal and Plant Health Agency (APHA), who lead diagnostic surveillance in Great Britain [19]. Through visual inspection of epidemic curves, cases were split into three distinct epidemic periods (EPs). EP1 covered 19 October 2021–9 February 2022 coinciding with a large peak in cases, EP2 covered 10 February 2022–16 August 2022 during which cases occurred infrequently and EP3 covered a large peak in cases between 17 August 2022 and 20 January 2023 (figure 1*a*).

To account for changes in each species’ relative abundance and HPAI case intensity across an EP, we weighted the weekly RADs by multiplying each cell value by the proportion of HPAI cases that occurred nationally within that week of the EP. We then sum these weekly weighted values to produce a single weighted relative species abundance for each grid cell (electronic supplementary material, figure S1). This weighting may be particularly necessary for species disproportionately abundant in part of the EP, e.g. migrants). Results using unweighted RADs are given in the supplementary material and were broadly consistent with weighted results (electronic supplementary material, data S3, figures S15−22 and tables S7−10).

To control for the effects of farm and poultry density, we used information on the location and stock numbers of premises obtained from the Great Britain Poultry Register (GBPR). As reporting to the GBPR is non-compulsory for flocks of <50 birds, we only used data from farms with 50 or more birds (19 680 farms). Farm locations and reported stock counts were rasterized across a regular grid at a resolution of 27 km^2^ to match wild-bird RAD data, giving a count of farms and the sum of stock numbers at the grid-cell level (electronic supplementary material, figure S1).

As alternatives to wild-bird RAD data, we also explored associations of premise cases with HPAIV-infected deceased wild birds and measures of alpha species diversity within each period. Spatial data on HPAIV-infected wild-bird carcasses was sourced from Empres-i [33]. Locations were rasterized across a regular grid at a resolution of 27 km^2^ with cell values representing total counts (electronic supplementary material, figure S2). Three measures of alpha diversity (species richness, Shannon’s index and Simpson’s index) were calculated using the package *abdiv* [34] at the grid-cell level for each EP using the weighted RADs for abundance (unweighted results in electronic supplementary material, data S3). While species richness represents a count of species with a weighted RAD >0, Shannon’s and Simpson’s indices also take account of evenness of abundance across species. Shannon’s index is a zero-bounded continuous measure (higher values inferring greater uncertainty of what species would be selected under random sampling), whereas Simpson’s index is a probability bounded at 0−1 (probability of selecting two individuals from different species under random sampling). In all cases, higher values infer greater diversity.

### (b) Primary data analysis

We fit spatial generalized additive models (GAMs; package *mgcv* [35]); with a binomial error family and a logit link function to test the effect of species abundance on premise cases at a 27 km^2^ grid-cell level. Our dependent variable (proportion of infected premises) was included as a two-column count matrix of infected and uninfected premises to perform weighted regression using the total number of premises within the grid cell as weights. Explanatory variables of weighted RADs and average stock number per premise were included as linear terms, which were scaled prior to model fitting. To account for spatial autocorrelation between neighbouring grid cells, we include a two-dimensional splined variable of latitude and longitude, fit with an isotropic smooth on the sphere. The default basis dimension (*k*) value of −1 and generalized cross-validation were used to estimate smoothness parameters. A separate model was fit for each species and EP where sufficient species abundance was present (sum of weighted RAD >1). Models that did not converge were removed from the model pool.

We also fit models where the count of HPAIV-infected wild birds and alpha diversity metrics at the grid-cell level were used instead of weighted relative species abundance. This resulted in a total of 398 independent models. To account for multiple testing, *p*-values were adjusted using two methods: (i) false discovery rate [36] and (ii) Holm–Bonferroni [37].

### (c) Post hoc analysis

We fit linear models (LMs) to assess group-level effects across our species-specific relative abundance associations. The linear slope coefficient of relative abundance from each of the species-specific independent models was used as the dependent variable, and the model was weighted by the reciprocal of the squared standard error of this slope coefficient. Categorical explanatory variables included the EP (1, 2 or 3), species grouping (see below) and their two-way interaction. Models of the same structure were run with varying coarseness of species grouping. Our coarsest grouping categorized species into three ecological sets of species*—landbirds*, *seabirds* and *waterbirds* (detailed classifications can be found in electronic supplementary material, S1), following classifications described in Geen *et al*. [38]. We then assessed subgroup-level patterns based on taxonomy and commonly used colloquial species groups. Groupings were chosen to reflect similarity in species behaviour, habitat use and phylogeny, which may influence a species’ likelihood of interacting with premises and being a HPAIV carrier. Some groupings (such as *seabirds*) were poorly represented as not all species present in Great Britain are modelled by eBird. We measured grouping coverage by comparing it against the British Ornithologists’ Union British list (excluding vagrants [39]; electronic supplementary material, table S5).

To assess the correlation between species-specific slope coefficients for the effect of species abundance across EPs, Pearson’s product moment correlation was used. We assessed group-level estimates of change between EP1 and EP3 using a general LM, where a change in slope coefficient from EP1 to EP3 was explained by species grouping. A separate model was run for each level of coarseness in species groupings.

All statistical analyses were performed with R 4.3.0 [40].

## 3. Results

To assess which wild-bird species abundance distributions are spatio-temporally associated with HPAI disease cases in captive avian premises across Great Britain, we modelled associations for 152 wild-bird species in three distinct EPs between October 2021 and January 2023 (figure 1). Spatial associations were assessed across 27 km^2^ grid cells, matching the coarsest spatial resolution of eBird status data products. We use the shorthand ‘case’ to refer to any captive avian premise in which one or more captive birds were confirmed to be infected with HPAIV via Polymerase chain reaction (PCR) and/or genetic sequencing and ‘premises’ to refer collectively to commercial, backyard and miscellaneous (e.g. rescue centres, zoos, etc.) premises where captive birds are kept. Most cases were reported from commercial premises (*n* = 216, 69.2%). EP1 and EP3 (19 October 2021–9 February 2022 and 17 August 2022–20 January 2023, respectively) covered large peaks in cases (*n* = 86 and *n* = 186, respectively) with a broad spread of cases throughout much of Great Britain. Conversely, EP2 (10 February 2022–16 August 2022) had lower case incidence (*n* = 40) and less ubiquitous spatial occurrence of cases, with regions such as northern and central-southern England having no cases despite relatively high farm density (figure 1*b*, electronic supplementary material, figure S1).

We assessed associations aggregated across groups of ecologically and phenotypically similar species (to minimize type-1 errors; electronic supplementary material, table S1), and at the individual species level (for full species-specific model coefficients see electronic supplementary material, data S1). Species-specific abundance associations with cases were obtained from models in which the effects of total poultry stock within the area were controlled for. Linear slope estimates for the effect of total poultry stock varied between models but were always positive (average slope estimates (95% CI): EP1 = 15.1 (14.6−15.5), EP2 = 23.1 (22.5−23.7), EP3 = 2.9 (2.8−3.1)), indicating areas with higher total poultry numbers had a greater proportion of infected premises during an EP.

### (a) Species groupings

**Figure 2.**
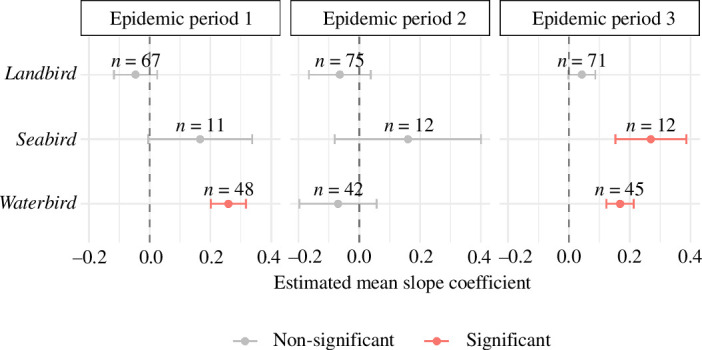
Estimated first-order group-level means for LM coefficients describing effects of weighted relative species abundance on probability of HPAI cases in premises. Estimated means derived from a GLM assessing group effects across EPs (EP1 = 19 October 2021–9 February 2022; EP2 = 10 February 2022–16 August 2022; EP3 = 17 August 2022–20 January 2023). *N* values above points indicate the number of species within each grouping. Error bars indicate the 95% confidence interval.

Our coarsest grouping considered three species groups: *landbirds*, *seabirds* and *waterbirds* (see electronic supplementary material, S1 for grouping details). *Waterbirds* showed consistent group-level significant positive associations with cases across EP1 and EP3 (figure 2; electronic supplementary material, table S2), indicating areas with higher *waterbird* abundance had a higher proportion of infected premises during these periods. *Seabirds*, in contrast, had positive associations in all EPs but only became significant within EP3 (figure 2, electronic supplementary material, table S2). However, *seabirds* had relatively poor species coverage within our analyses being predominantly represented by gull and tern species. *Landbirds* showed no significant positive association in any EP (figure 2, electronic supplementary material, table S2), and this was driven in part by a lack of consistency in the direction of associations between individual species or species subgroups.

### (b) Landbirds

**Figure 3.**
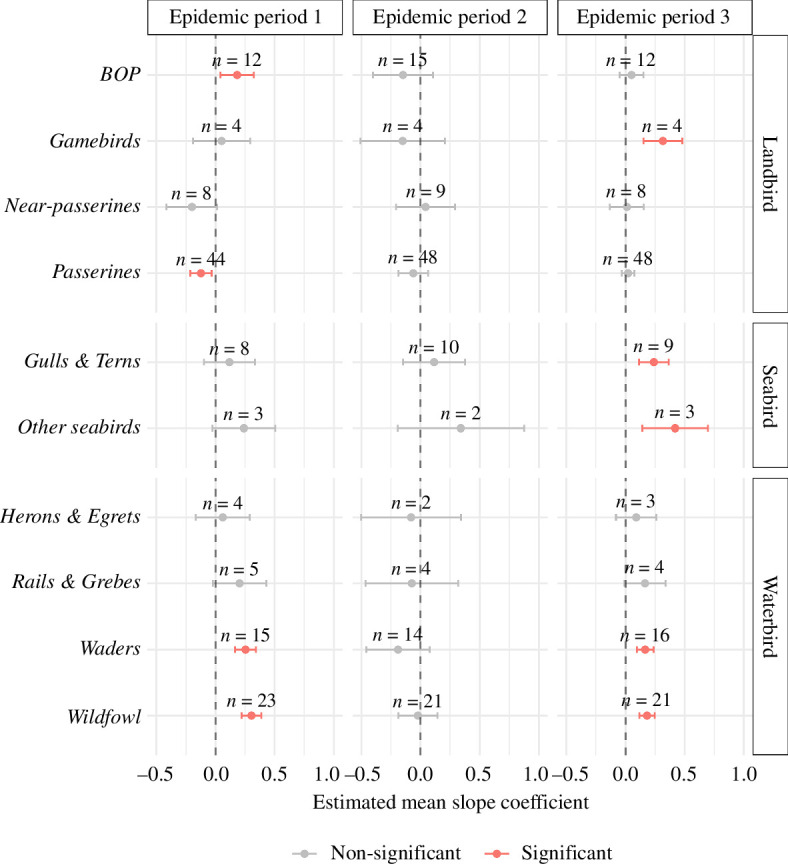
Estimated second-order group-level means for LM coefficients describing effects of weighted relative species abundance on the probability of HPAI cases in premises. Estimated means derived from a GLM assessing group effects across EPs (EP1 = 19 October 2021–9 February 2022; EP2 = 10 February 2022–16 August 2022; EP3 = 17 August 2022– 20 January 2023). *N* values above points indicate the number of species within each grouping. BOP refers to the *Birds of Prey* grouping.

Despite no significant association for *landbirds* in any EP, significant associations were found in some subgroupings (figure 3, electronic supplementary material, figure S3,, table S3 and table S4). Infected premises during EP1 had a significant positive association with *birds of prey* (families *Strigiformes, Accipitridae* and *Falconidae*), although this was limited to *diurnal raptors* (families *Accipitridae* and *Falconidae*)*,* which also had a significant positive association during EP3. *Passerines* (order Passeriformes) showed a significant negative association but there was inconsistency amongst its subgroups; subgroupings such as *tits* (family *Paridae*), *flycatchers* and *chats* (family *Muscicapidae*), and *thrushes* (family *Turdidae*) showed significant negative associations, whereas *sparrows* (family *Passeridae*) had a significant positive association. A significant positive association with *sparrows* was also observed during EP3.

During EP3, *gamebirds* (family *Phasianidae*) gained a significant positive association (figure 3, electronic supplementary material, table S3). This effect was the strongest amongst *non-native gamebirds*, which are bred and released in large numbers during the late-summer and early-autumn period that is included within EP3 [41] (electronic supplementary material, figure S3 and table S4). In *native gamebirds*, the significant positive association was primarily driven by Grey Partridge *Perdix perdix*, which are also bred and released in some areas as part of species recovery projects, albeit in far smaller numbers ([42]; electronic supplementary material, figure S4). As was the case in EP1, *passerine* subgroupings displayed inconsistency; *flycatchers* and *chats* had a significant negative association, whereas other groups such as *buntings* (family *Emberizidae*) and *larks, pipits* and *wagtails* (families *Alaudidae* and *Motacillidae*) had a significant positive association (electronic supplementary material, figure S3 and table S4).

There was no significant association for near*-passerines* (families *Columbidae*, *Picidae*, *Psittaculidae* and *Alcedinidae*) during any EP, nor were there any significant associations amongst any of the *landbird* subgroupings during EP2 (figure 3, electronic supplementary material, figure S3, table S3 and table S4).

### (c) Waterbirds

*Wildfowl* (order Anseriformes) showed significant positive associations across EP1 and EP3 (figure 3; electronic supplementary material, table S3). All subgroupings had positive associations, although not all are significant (electronic supplementary material, figure S3 and table S4). *Dabbling ducks* (subfamily *Anatinae*) were significant in both periods, whereas *diving ducks* (subfamily *Aythyinae* and tribe Mergini) were only significant in EP3 (electronic supplementary material, figure S3 and table S4). Other subgroupings were non-significant; however, there were significant positive species-specific associations in highly abundant, and therefore potentially important, species such as Greylag Goose *Anser anser* and Mute Swan *Cygnus olor* (electronic supplementary material, figure S4). *Waders* (suborders Charadrii and Scolopaci) also showed significant positive associations in both EP1 and EP3 (figure 3; electronic supplementary material, table S3), driven by subgroups *plovers* and *sandpipers* (families *Charadriidae* and *Scolopacidae*) in both periods (electronic supplementary material, figure S3 and table S4). Across the other subgroups*,* namely *herons* and *egrets* (family *Ardeidae*) and *rails* and *grebes* (families *Rallidae* and *Podicipedidae*), there were no significant associations during any of the EPs (figure 3; electronic supplementary material, figure S3).

### (d) Seabirds

Despite having a positive association across all EPs, *seabirds* were only found to have a significant association in EP3. However, this broad grouping had a poorer species coverage across modelled eBird species compared with *waterbirds* and *landbirds* (electronic supplementary material, table S5), with many of the more substantial subgroupings not assessed. *Seabird* groups such as Auks (family *Alcidae*), Petrels and Shearwaters (family *Procellariidae*), as well as focal species such as the Northern Gannet *Morus bassanus* and Great Skua *Stercorarius skua* (which have been linked to mass outbreaks in wild birds in Great Britain [10,15]), were not represented.

Here, *seabirds* are therefore predominantly represented by the *gulls* and *terns* (family *Laridae*) subgrouping, which had a significant positive association in EP3 (figure 3; electronic supplementary material, table S3). This significant association also remained when this subgrouping was split into *gulls* (subfamily *Larinae*) and *terns* (subfamily *Sterninae*) separately (electronic supplementary material, figure S3 and table S4). The remaining three seabird species assessed were grouped into *other seabirds,* which was also significantly positive in EP3 (figure 3; electronic supplementary material, table S3). This grouping only represents a limited range of seabird species (mainly divers), and the only significant subgrouping was *divers* (family *Gaviidae*) in EP3 (electronic supplementary material, figure S3 and table S4).

### (e) Species-specific associations

**Figure 4.**
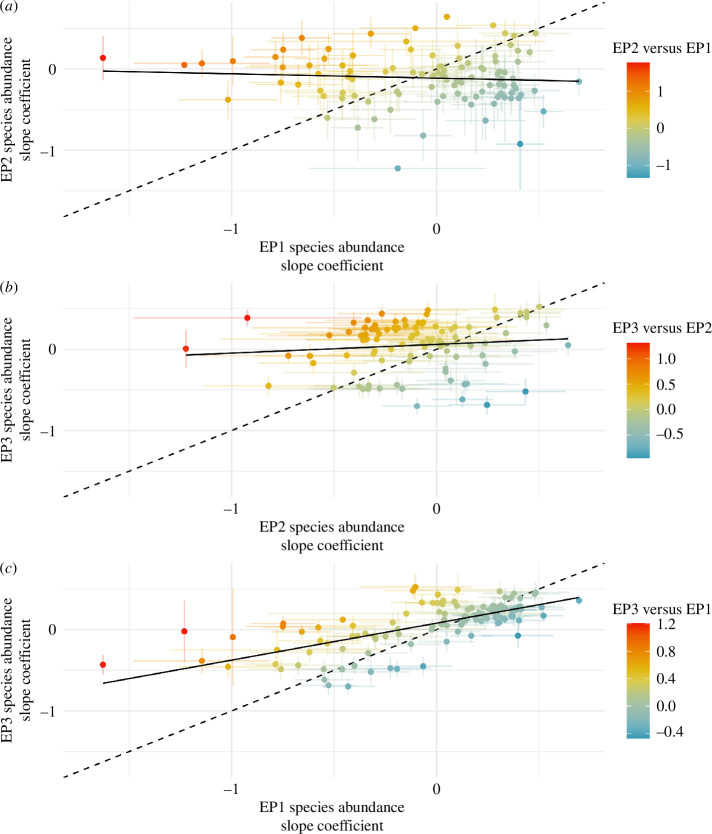
Change in species-specific slope coefficient for the effect of weighted relative species abundance on case probability in premises across Great Britain. (*a*) EP2 compared with EP1. (*b*) EP3 compared with EP2. (*c*) EP3 compared with EP1. The dashed black line has a slope of 1 and indicates the relationship if there was no change between EPs. Solid black line is the actual line of best fit through points based on a LM. Error bars around points indicate standard error.

For species assessed in multiple EPs, species-specific associations were poorly correlated across subsequent periods (EP1–EP2: cor = −0.075 (95% CI: −0.264–0.119), d.f. = 102, *p* = 0.447; EP2–EP3: cor = 0.113 (95% CI: −0.085–0.303), d.f. = 98, *p* = 0.263; figure 4*a,b*), indicating a potential change in viral dynamics and potential host species during the summer period of low HPAI incidence compared with the winter periods of high incidence. Conversely, species-specific associations were significantly correlated between EP1 and EP3, which are both characterized by large peaks in cases (EP1–EP3: cor = 0.691 (95% CI: 0.581−0.776), d.f. = 112, *p* < 0.001; figure 4*c*).

**Figure 5.**
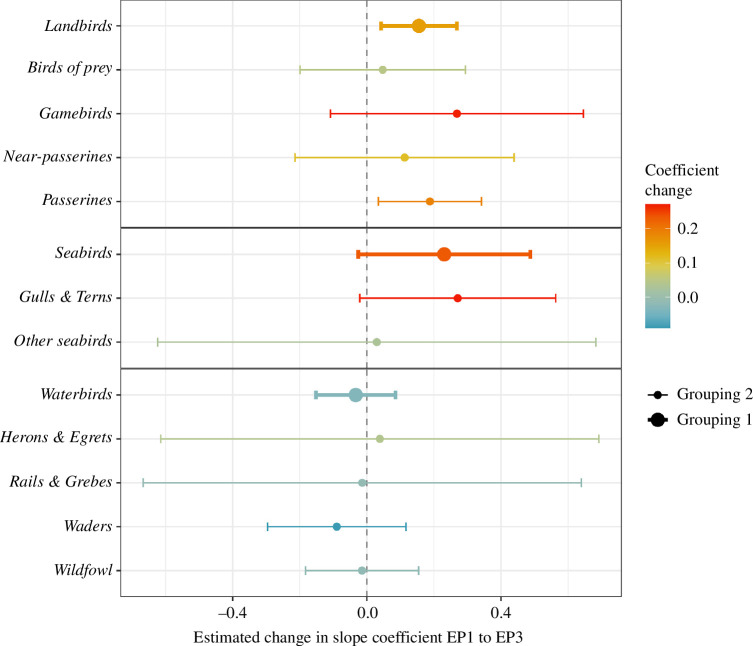
Estimated means for change between EPs in the LM coefficients describing effects of weighted relative species abundance on the probability of HPAI cases in premises. Estimated means derived from a GLM assessing group effects (separate model for grouping 1 and grouping 2). Change is calculated as slope coefficient in EP3 minus slope coefficient in EP1. Bold text and thicker error bars indicate models under the coarsest species grouping (grouping 1).

Despite a significant positive correlation between species abundance slope coefficients in EP1 and EP3, there were also distinct outliers where coefficients shifted between EPs. These outliers are of potential interest, as they may represent changes in typical viral host range. Both *landbirds* and *seabirds* had significant increases in effect sizes in EP3, as did subgroups *gulls* and *terns* and *passerines*. All other *landbird* and *seabird* subgroupings had non-significant increases. *Waterbirds*, and its subgroupings, predominantly showed small non-significant decreases (figure 5, electronic supplementary material, table S6).

Due to the potential for type 1 errors (i.e. false positives) with multiple testing across many independent models, single-species relationships may risk overinterpretation of model results, as some significant positive spatial associations may arise by chance alone. Without correcting for multiple testing, significant positive associations were found in 41 (32.3%), 8 (6.2%) and 59 (45.7%) species across the three EPs, respectively (electronic supplementary material, figure S4). To account for this, *p*-values can be adjusted. After applying the false discovery rate method (FDR [36]), significant positive associations were found in 37 (29.1%), 1 (0.8%) and 54 (41.9%) species across the three EPs, respectively (electronic supplementary material, figure S4). The more conservative Holm–Bonferroni [37] adjustment method left only 22 (17.3%), 1 (0.8%) and 27 (20.9%) species significant across the three periods, respectively (electronic supplementary material figure S4).

Significant negative associations were also found in 25 (19.7%), 5 (3.9%) and 21 (16.3%) species across the three EPs, respectively (electronic supplementary material, figure S5 ; FDR: 23 (18.1%), 0 (0%), 20 (15.5%); Holm–Bonferroni: 10 (7.9%), 0 (0%), 16 (12.4%)). Negative associations were, however, generally of lower magnitude and were also relatively uncommon in EP1 and EP3, where we had greater statistical power due to larger numbers of HPAI cases.

### (f) Associations with wild-bird cases

Our methods do not require data on viral detection in wild birds. To explore the efficacy of using wild-bird abundance data alone compared with using data on confirmed infection in wild birds, we also tested whether PCR-diagnosed HPAIV-infected wild-bird carcasses were associated with infected premises. The number of confirmed infections in wild birds fell across the EPs, with 425, 163 and 96 reported, respectively (Empres-I database; electronic supplementary material, figure S6); however, this was likely a substantial underrepresentation of wild-bird infection rates across these periods. Infected wild-bird cases were positively associated with infected premises across all EPs, but the association was only significant within EP1 and EP2 (EP1 (winter 2021/2022): estimate = 0.050 ± 0.019 s.e., *p* = 0.009; EP2 (spring/summer 2022): estimate = 0.641 ± 0.112 s.e., *p* < 0.001; EP3 (autumn/winter 2022/2023): estimate = 0.051 ± 0.123 s.e., *p* = 0.679; electronic supplementary material, figure S4); however, the significance in EP1 is lost if Holm–Bonferroni multiple-testing correction is applied.

### (g) Association with species diversity

We also considered it possible that a greater diversity of species could confer greater incursion risk if the presence of different long-distance vectors, local amplifiers and bridging species is required to drive incursions into farms. We found species richness to have significant positive associations in EP1 and EP3. However, other metrics of alpha diversity tested (Shannon’s and Simpson’s indices), which account for evenness of abundance between species, had no significant association in either EP. Within EP2, all three metrics were found to have significant negative associations (electronic supplementary material, figure S11 and data S1).

### (h) Model performance

Species-specific models fitted to EP1 and EP3 generally performed better than models fitted to EP2, with higher median deviance explained in these periods on average (EP1 = 0.341 ± 0.002 s.e., *n* = 127; EP2 = 0.242 ± 0.002 s.e., *n* = 130; EP3 = 0.373 ± 0.002 s.e., *n* = 129; electronic supplementary material, figure S7). The poorer model performance in EP2 is likely driven by the comparatively low number of cases of infected premises (*n* = 40; electronic supplementary material, figure S6), combined with a weaker spatial clustering of cases. This poor model performance is also likely to be a major contributing factor to the greater dissimilarity between species-specific associations during EP2 across closely related species.

## 4. Discussion

Gaining an understanding of which wild species may be facilitating viral incursions into well-monitored populations can be extremely challenging, particularly in dynamic, multi-host systems. Our study demonstrates how publicly available citizen science outputs of wild species abundance can be a powerful tool for exploring spatio-temporal associations between wild species abundance and disease cases in focal, well-monitored populations. Specifically, we assessed the spatial association between avian abundance distributions from eBird and cases of HPAI in poultry premises across Great Britain. Across three distinct EPs between October 2021 and January 2023, 103 species had a significant association in at least one period, and several groups of ecologically similar species were also found to be significant. There was broad consistency between both major infection waves (EP1 and EP3), with groups such as *wildfowl* having consistent significant group-level positive associations with infected poultry premises, alongside some changes at both the group and species-specific level. In contrast, during the summer period where infected premises were uncommon, our models performed comparatively poorly, failed to find consistent effects across similar species and wild-bird abundance distributions had a less clear association.

Despite our method not relying on HPAIV surveillance in wild birds or any prior assumptions for susceptibility, many of the associations we have demonstrated are commensurate with our existing understanding of which avian species play a greater role in the dissemination of HPAIVs. Although more than 220 wild-bird species have tested positive for HPAIV globally since 2020 [43], the primary wild-bird species typically associated with maintenance and spread belong to the orders Anseriformes and Charadriiformes, with other less commonly implicated orders usually also associated with aquatic habitats [44]. However, large-scale, systematic studies of HPAIV are largely lacking in other Orders, despite the limited studies that have been undertaken finding widespread prevalence (albeit often at low levels) [45]. In this study, *waterbirds* were consistently found as significant predictors in both periods, covering large peaks in cases (EP1 and EP3) with little change in effect sizes.

We also found that the group *seabirds* showed significant increases in effect sizes during EP3. This matches a widely noted step-change in host range during the summer of 2022 across Europe, where major disease outbreaks were recorded in many seabird species at a scale previously not seen [16,46,47]. Our results for *seabirds* are, however, largely driven by *gulls* and *terns* that have more extensive terrestrial distributions than many other seabird species and can often be found terrestrially in the UK year-round. Many other species of *seabirds* across the wider group may, therefore, be unlikely to have such correlation with outbreaks in captive avian premises. One subgroup we were able to assess and did find significant associations with was *divers*, which was unexpected due to their relatively sparse distribution in much of the UK and life history that would mean direct contact with captive avian premises being unlikely. This may simply be a result of collinearity in distributions between divers and other species. For example, during EP3, we see moderate positive correlations between the abundance of diver species and some gull and tern species (electronic supplementary material, S2 Data, Pearson’s correlation coefficients 0.5−0.7). However, divers have been shown to have seroprevalence for AIV in other regions, albeit at low levels [48], and subgroups such as *gulls* are likely to interact with other seabirds through the breeding season. They may, therefore, play a key role as a bridge species between disease outbreaks in isolated seabird colonies and terrestrial bird populations.

Many of the most conspicuous mass-mortality events within seabirds occurred within species that we could not assess here due to their omission from eBird-modelled species. This included species such as Northern Gannet and Great Skua, where significant proportions of breeding populations were lost [10,15]. However, these species tend to be present in the UK only in the breeding season and are highly restricted to small islands and coastal edges when not at sea. They are, therefore, unlikely to have abundance distributions that correlate with outbreaks in premises.

We also found significant group-level associations of premise cases with abundance of some passerine groupings, such as sparrows during EP1 and EP3. While there is little existing evidence of passerines having a major role as a maintenance reservoir host or bridge species facilitating virus transmission, this finding may indicate that greater targeted surveillance of some passerine species may be worthwhile, particularly those known to frequent poultry premises. Furthermore, while passerines tend to show low test positivity rates for HPAIV [17,49] and may, therefore, have limited capacity as biological vectors of the virus (i.e. virus replicates inside the wild host), they may also act as mechanical transporters via fomite (e.g. virus carried externally on feet or feathers). The external swabbing of small passerines in the USA during previous outbreaks found no support for mechanical transport potential via fomites [50]. However, small sample sizes together with large populations typical of small passerines lead to low confidence in prevalence. If passerines are acting as sources of (mechanical or biological) transmission between captive and wild birds, many species are small and can be difficult to exclude from poultry premises or adjacent habitats via biosecurity measures unless bird exclusion measures are of high standard and well-maintained, and therefore may pose a significant ongoing risk of virus transmission.

Of particular note was the association of cases with free-living *non-native gamebirds* during EP3. Although exact numbers are undefined, tens of millions of non-native gamebirds are reared and released in the UK each year to bolster feral breeding populations (breeding pop. est. ~4.4 million [51]); for the purpose of gamebird shooting [41]. Both Common Pheasant *Phasianus colchicus* and Red-legged Partridge *Alectoris rufa*, the two non-native gamebirds released in the highest numbers annually, had species-specific positive associations during EP3. While eBird-modelled abundances are not a direct measure of gamebird release locations, it is expected that there would be a high degree of correlation between the two as released birds typically only disperse a short distance from release sites (<1 km for 90−95% of released pheasants) and have low survival rates following release (~15% at the end of shooting season [52]). Furthermore, significant numbers of Mallard *Anas platyrhynchos* (a native dabbling duck) are also released for shooting within this period (estimated ~2.6 million per year compared with ~31.5 and ~9.1 million Common Pheasant and Red-legged Partridge, respectively [41]); and also had a significant positive association during EP3.

The role of reared and released birds in HPAIV maintenance and spread is controversial. Despite cases within gamebird hatcheries and rearing premises [53,54], there remains a lack of conclusive evidence for the role of released gamebirds in the spread of HPAIV. The likelihood of HPAIV incursions into the gamebird sector was considered low based on egg and chick movements [53] but higher during ‘catching-up’ (when surviving released gamebirds may be re-caught after cessation of the shooting period [28]). However, the risk of virus spread from free-roaming gamebirds to wild birds post-release was deemed high and has the potential to contribute to viral maintenance in other wild-bird populations, ultimately leading to increased infection pressure [55]. Furthermore, experimental *in vivo* inoculation with clade 2.3.4.4b H5N6 has shown that the Common Pheasant is capable of both acquiring HPAIV and subsequently transmitting to poultry [56]. Similar outcomes were seen following experimental infection of pheasants with clade 2.3.4.4b H5N1 and H5N8 viruses, with pheasants being more susceptible to infection than Red-legged Partridges [57]. The significant positive associations with *non-native gamebirds* that we identify here support calls for increased surveillance of this group to more robustly assess the potential role of non-native gamebirds in facilitating HPAIV transmission, particularly following release.

Our lack of any significant association with the subgroup *Pigeons* and *Doves* (family *Columbidae*) or any specific individual species therein is of note, because columbids can be highly abundant in and around poultry premises. Species in this family can become infected with HPAIV [58,59], but in general appear to show high resistance to infection and low viral shedding [60]. Our results would seem to support that this may limit their role in virus transmission through limiting their ability to acquire HPAIV from poultry, contaminate farm environments or directly infect poultry. It may also indicate that biosecurity measures in the UK have been generally effective at preventing transmission pathways involving columbids.

Spatial associations between wild-bird abundance and HPAI cases in premises can help steer targeted surveillance efforts to find species that continue to facilitate viral incursions (either directly onto premises or indirectly by acting as local amplifiers). However, such associations cannot prove causative relationships and may arise from chance alone or due to collinearity between RADs of different species. Indeed, many moderate (0.5−0.7 or −0.5 to −0.7) and high (>0.7 or <−0.7) Pearson’s correlation coefficients were present between our RADs (electronic supplementary material, S2 Data). This may cause the few negative associations found in this study, as there are few mechanisms through which a higher abundance of a single wild-bird species would drive lower disease incidence across premises. While such species could be outcompeting others with generally higher viral incidence, and consequently lowering incursion risk, we do not expect this to be likely.

Despite these limitations, our approach benefits from the ability for rapid deployment without the requirement for extensive prior surveillance of disease in all potential host species, which is often financially and logistically prohibitive. Even in high-income countries such as the UK, logistical constraints such as the reporting, triage, collection and transport of carcasses, as well as legal restrictions on sample handling, can severely limit testing capacity. Notably, we saw a marked decline in HPAIV-infected wild birds across our three EPs despite an increase in premise cases (electronic supplementary material, figure S6). Indeed, insufficient HPAIV-positive wild birds may be the cause for the lack of predictive power observed in EP3, where they did not significantly predict premise cases.

Our research shows that the use of publicly available wild-bird abundance and distribution data can complement surveillance in wild birds to help identify species that could be prioritized for testing in close to real time. This could help to target limited surveillance resources and to monitor potential changes in infected wild species even in the absence of high mortality. Rapid deployment may also aid in risk mitigation for incursions onto premises, by enabling improved biosecurity measures to be put in place with minimal delay that are better tailored towards higher-risk species. The use of eBird data is particularly valuable because predictions are made at the global scale, at relatively high resolution and are free to access for over 2000 species [31]. However, continued efforts may be needed to improve capacity building in regions where the requisite citizen science data may be lacking, and species abundance distributions cannot be accurately modelled.

Despite our analyses largely relying on publicly available data, we controlled for avian livestock and farm density using poultry distribution datasets that are not publicly available in Great Britain. Controlling for avian livestock density may be somewhat achievable globally with The Gridded Livestock of the World 3 ([61]; ~10 km^2^ resolution), but farm density data may only be available for some countries. We also lack sufficient data on how biosecurity (in terms of preventing wild-bird incursion) may vary across premises, and whether there are significant spatial patterns in this across Great Britain. Improved understanding of these potential biases would enable more accurate spatio-temporal associations between wild-bird abundance and HPAI case risk in premises.

## 5. Conclusions

Publicly available wild-bird abundance and distribution predictions developed using citizen science bird-counting initiatives, such as those offered by eBird, can be a powerful tool in helping to screen for potential drivers of wild-bird-mediated virus incursions into closely monitored populations such as poultry, livestock, endangered species or humans. Here, higher abundance in avian groups such as wildfowl (ducks, geese, etc.) was found to be consistently associated with higher HPAI incidence in premises. Some avian species groups, however, became more important in the most recent EP, perhaps linked to a change in viral host range, or species-specific drivers such as *non-native gamebirds* only being associated with HPAI in premises during the period coinciding with their mass release. These associations may help guide future targeted mass surveillance and aid understanding of the changing host range of HPAIV as it continues to adapt and spread in wild-bird populations.

## Data Availability

Analysis code and model output results are available on Dryad [61]. eBird species abundance distributions are publicly available directly from eBird via [62]. Premise HPAIV outbreak data and Great British Poultry Register data are protected and cannot be publicly shared due to GDPR, requiring data-sharing agreements. These data can be requested by directly contacting the Animal and Plant Health Agency. Supplementary material is available online [63].
